# LigDig: a web server for querying ligand–protein interactions

**DOI:** 10.1093/bioinformatics/btu784

**Published:** 2014-11-29

**Authors:** Jonathan C. Fuller, Michael Martinez, Stefan Henrich, Antonia Stank, Stefan Richter, Rebecca C. Wade

**Affiliations:** ^1^Molecular and Cellular Modeling Group, Heidelberg Institute for Theoretical Studies (HITS), 69118 Heidelberg, ^2^Center for Molecular Biology Heidelberg University (ZMBH), DKFZ-ZMBH Alliance, 69120 Heidelberg, Germany and ^3^Interdisciplinary Center for Scientific Computing (IWR), 69120 Heidelberg, Germany

## Abstract

**Summary:** LigDig is a web server designed to answer questions that previously required several independent queries to diverse data sources. It also performs basic manipulations and analyses of the structures of protein–ligand complexes. The LigDig webserver is modular in design and consists of seven tools, which can be used separately, or via linking the output from one tool to the next, in order to answer more complex questions. Currently, the tools allow a user to: (i) perform a free-text compound search, (ii) search for suitable ligands, particularly inhibitors, of a protein and query their interaction network, (iii) search for the likely function of a ligand, (iv) perform a batch search for compound identifiers, (v) find structures of protein–ligand complexes, (vi) compare three-dimensional structures of ligand binding sites and (vii) prepare coordinate files of protein–ligand complexes for further calculations.

**Availability and implementation:** LigDig makes use of freely available databases, including ChEMBL, PubChem and SABIO-RK, and software programs, including cytoscape.js, PDB2PQR, ProBiS and Fconv. LigDig can be used by non-experts in bio- and chemoinformatics. LigDig is available at: http://mcm.h-its.org/ligdig.

**Contact:** jonathan.fuller@h-its.org, rebecca.wade@h-its.org

**Supplementary information:** Supplementary data are available at *Bioinformatics* online.

## 1 Introduction

There has been growing discussion of the ‘data deluge’ and the perceived problems surrounding it, namely how to store, process and visualize data, and how to combine multiple heterogeneous data sources. For many working in the cross-disciplinary environment of systems biology research, the challenge of combining these data sources can be great, but the rewards of doing so can be even greater. One of the hurdles facing researchers is the wide variety of data sources available. Therefore, computational tools to assist in the analysis of this data are essential. Tools to assist the modeling of biochemical networks, such as SYCAMORE (Weidemann *et al.*, 2008) or MetNetMaker (Forth *et al.*, 2010), can simplify the construction of metabolic models, although most require knowledge of database identifiers for the biological entities of interest. Such tools show the utility of integrating data from several sources and simplifying the handling of multiple database identifiers. LigDig uses the strategy of accessing multiple data sources and assisting the user to interpret results that might not be possible by visiting a single data source alone. Additionally, LigDig assists users in performing basic manipulations of protein structures that can help researchers to gain new insights into their system of interest.

## 2 Design

LigDig was developed to assist a user with a set of example questions that might be encountered in a typical systems biology workflow. Aware that new examples could be encountered during software development, we used a modular design that allows component reuse and easy inclusion of new features. An overview of the workflow for the individual tools is given in Supplementary Figure S1. We developed most functionality using Python 2.7, since many powerful toolkits [Biopython (Cock *et al.*, 2009), Open Babel (O’Boyle *et al.*, 2011), LibSBML (Bornstein *et al.*, 2008)] are available. The front-end was developed using Play Framework 2, a Model-View-Controller framework allowing code to be written in Java or Scala, with templating of HTML pages using Scala templates. Long running jobs can be provisioned through the Akka framework (an actor-based concurrency model), which allows the website to remain responsive to users. Twitter bootstrap (a front-end web development framework) was used as a way to provide user interface elements that can be viewed on mobile and desktop browsers.

## 3 Examples

LigDig includes examples for each tool. These provide new users with an overview of its functionality and usage. Here, we briefly discuss two examples. When user input to the tools is required, the text is shown in *italic*.

### 3.1 How and where can information be found for the compound fructose 1,6-bisphosphate?

With the example query ‘fructose 1,6-bisphosphate’, we find no compounds in the ChEMBL database (Gaulton *et al.*, 2012). We do not believe that this is a deficiency of the ChEMBL database, rather a reflection on the typical user of ChEMBL, who is not likely to provide an InChI (Heller *et al.*, 2013). Re-querying ChEMBL using ‘fructose 1,6-diphosphate’ yields the desired compound (CHEMBL97893); however, this is a sodium salt of the compound, and the search does not find (CHEMBL1089962) which is stored in ChEMBL as ‘Fructose-1,6-Diphosphate’. Similarly, searching using ChemHITS (Golebiewski *et al.*, 2009), a user could find a compound in SABIO-RK (SABIO-Compound-ID: 20960) (Wittig *et al.*, 2012), but not in ChEBI (Degtyarenko *et al.*, 2008) or KEGG (Kanehisa and Goto, 2000). Additionally, in the case of the compound found, no 2D chemical structure is provided; thus, it is difficult to check that the correct compound is found. LigDig provides a solution to the compound searching problem, by searching the PubChem database using a relatively broad search term. *The user inputs text that describes the compound of interest, e.g. fructose 1,6-bisphosphate or similar, LigDig then returns several possible results*. These results are grouped by the atomic connectivity. The user is then shown a table with the possible compounds, including their 2D structures, as well as a disambiguation to other databases, which is provided through the UniChem tool. For the search ‘fructose 1,6-bisphosphate’, LigDig identifies nine possible compounds, of which six compounds are classed into two groups of identical connectivity (size four and two, respectively). The group containing four PubChem chemical identifiers (CIDs: 172313, 10267, 445557, 718) contains links to the two correct ChEMBL compounds: CHEMBL97893 and CHEMBL1089962. LigDig enables the user to easily search for their compound of interest. *The user can now choose to follow links in the results table directly to the original data source, or to continue with further investigations in LigDig by using checkboxes or buttons to submit to further analysis tools.* LigDig also provides the number of structures in the Protein Database (PDB; Berman *et al.*, 2002) which contain the compound of interest.

### 3.2. Which proteins does my ligand inhibit and why?

**Fig. 1.**
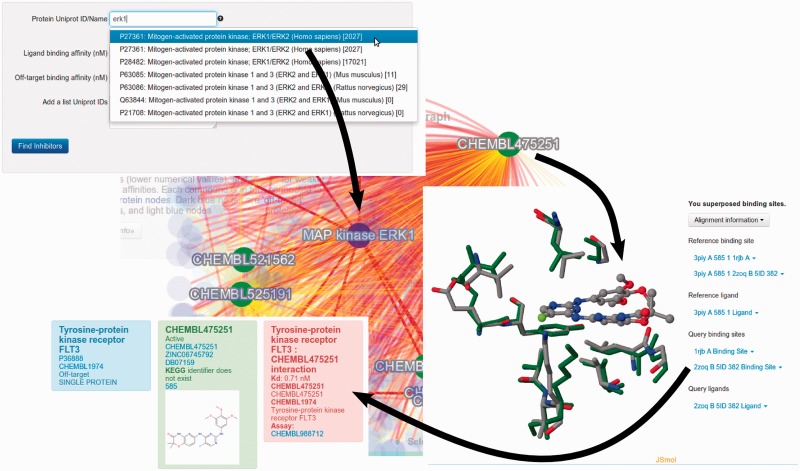
Illustration of the output of the LigDig web server for the example ‘*Which proteins does my ligand inhibit, and why?’*. See text for details

Consider the case of the Mitogen-activated protein (MAP) kinase signaling network. A user interested in the human protein ERK1 (Uniprot P27361), would use the ‘Find inhibitor’ tool. *They would enter the uniprot identifier if already known. Otherwise the user can do a text search which is autocompleted using a typeahead javascript. The user also needs to select two cutoff values to define which compounds are considered to bind to the target and to off-targets; here, we use 10 nM and 100 000 nM, respectively.* The tool identifies the compound CHEMBL475251, which using the UniChem web service (Chambers *et al.*, 2013) is disambiguated as the drug Tamatinib (see Fig. 1). There are currently two protein structures containing this compound available (PDB: 3piy and 3fqs). The cytoscape.js (an open-source Javascript graph theory visualization library) and tabular views of the search show that Tamatinib can also bind to the FLT3 tyrosine protein kinase receptor. Clicking on the edge linking CHEMBL475251 and FLT3 shows the binding affinity of this interaction. *LigDig enables the user to investigate the structural basis for this binding using the ‘Superpose ligand binding sites’ tool by entering the PDB codes provided for ERK1 (2zoq), FLT3 (1rjb) and a structure containing Tamatinib, here a Bruton’s tyrosine kinase (3piy).* Since there is currently no structure of Tamatinib bound to FLT3 in the PDB, superposition of the latter two structures gives a suggestion for the probable binding pose of Tamatinib in the FLT3 binding site. If the user is interested in the structural basis for Tamatinib binding to ERK1 and FLT3, the user can also superpose ERK1 onto the Bruton’s tyrosine kinase structure at the same time. Comparisons of the binding site to the reference structure, here FLT3, can be displayed, along with root-mean-square deviation values, in this case indicating that the binding sites of these protein kinases are highly conserved.

## Supplementary Material

Supplementary Data
