# Supplementary material for: LigDig: a web server for querying ligand–protein interactions
Source: Bioinformatics. 2014 Nov 29;31(7):1147–9. doi: 10.1093/bioinformatics/btu784 (PMC4382906; doi:10.1093/bioinformatics/btu784)
Supplement: Supplementary Data [file supp_31_7_1147__index.html]

LigDig: a web server for querying ligand-protein interactions — LigDig: a web server for querying ligand–protein interactions — LigDig: a web server for querying ligand–protein interactions — Supplementary Data 

# LigDig: a web server for querying ligand–protein interactions

## Supplementary Data

files

**Files in this Data Supplement:**

- Supplementary Data - pdf file
