# Supplementary material for: LigDig: a web server for querying ligand–protein interactions
Source: Bioinformatics. 2014 Nov 29;31(7):1147–9. doi: 10.1093/bioinformatics/btu784 (PMC4382906; doi:10.1093/bioinformatics/btu784)
Supplement: Supplementary Data [file supp_btu784_bio_ligdig_supporting_info.pdf]

Application Note – Systems and structural biology

LigDig: a web server for querying ligand-protein interactions

Jonathan C. Fuller[1,*], Michael Martinez[1], Stefan Henrich[1], Antonia Stank[1], Stefan Richter[1] and Rebecca C. Wade[1,2,*]

[1]Molecular and Cellular Modeling Group, Heidelberg Institute for Theoretical Studies (HITS), 69118 Heidelberg, and [2]Center for Molecular Biology Heidelberg University (ZMBH), DKFZ-ZMBH Alliance, 69120 Heidelberg, Germany

Received on XXXXX; revised on XXXXX; accepted on XXXXX

Associate Editor: XXXXXXX

© Oxford University Press 2005

1 WORKFLOW

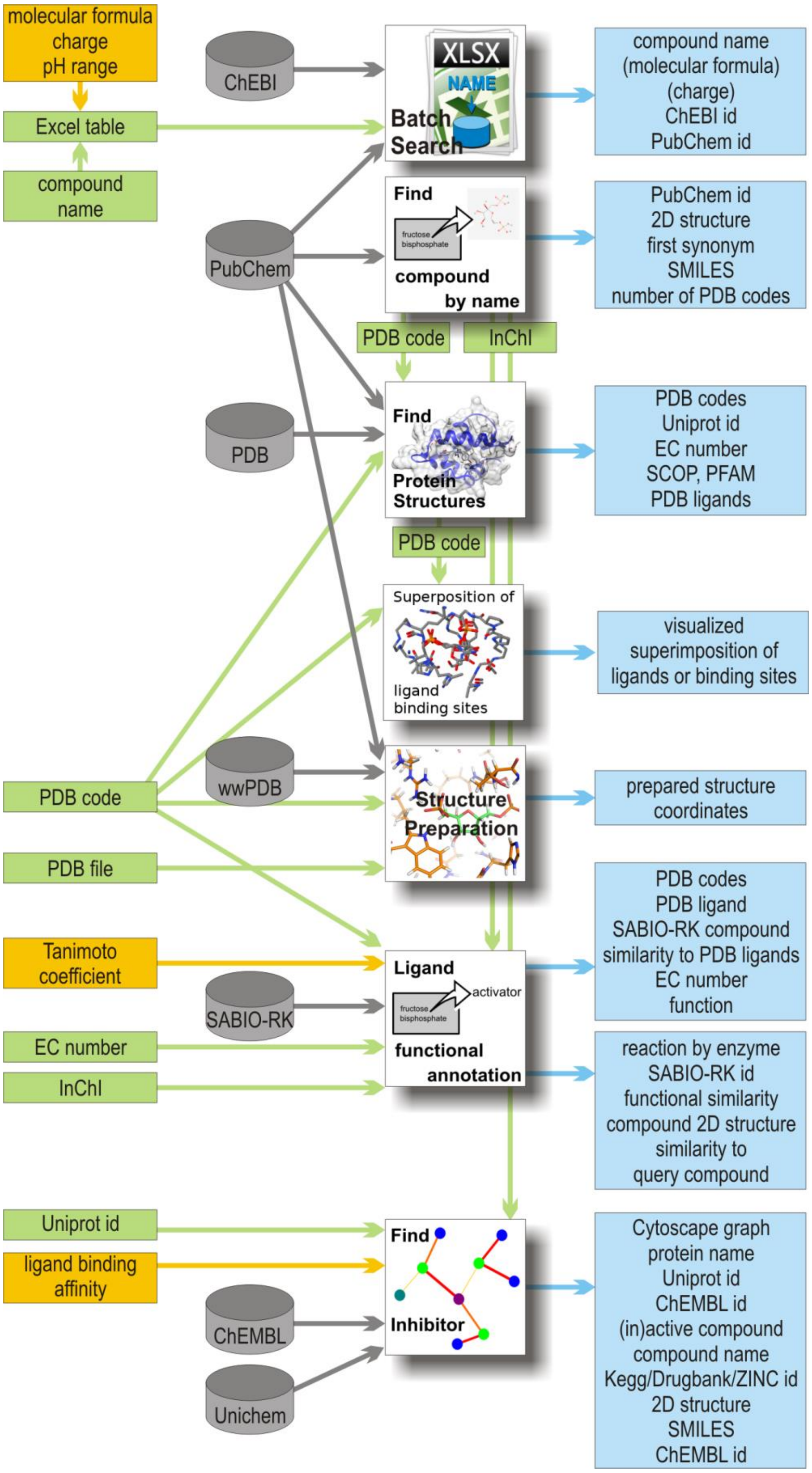

Fig. 1. A workflow showing the modularity of LigDig component tools, what input data they require (green: mandatory, orange: optional), which databases they access (grey), and what output data they produce (cyan). Possibilities to link output data from one tool to another are shown with green arrows from the tool icons.

© Oxford University Press 2005

2 MOTIVATION, FUNCTIONALITY, AND DESIGN

2.1 Find compound by name

Motivation: When constructing a model of glycolysis for which much of the data comes directly from reading and interpreting the literature, the user may start only with a compound name. Disambiguation of compound names can be rather challenging for automated methods. For example, as of July 2014, searching the ChEMBL web interface with 'fructose 1,6-bisphosphate' yields no results whilst 'fructose 1,6-diphosphate' can find a compound. A typical user could normally identify the correct compound from a short list of possibilities. This would allow the user to easily disambiguate the name of a compound to a unique database identifier, which can be a useful starting point for further queries.

Functionality: An initial query of compound names in the PubChem Compound database (Bolton, 2008) is made through the Power User Gateway (PUG) interface. The results are aggregated and displayed as a table. The results are similar to the text search from the PubChem Compound website, but additionally the compounds are grouped based on their connectivity (using the first 14 letters of the InChI-Key)(Heller *et al.*, 2013). The compound search also provides the canonical SMILES of the elements that are used to retrieve the list of PDB codes from the RCSB PDB database (Berman *et al.*, 2002). The result table contains the total number of crystal structures containing the compound, which can be further analysed by the tool *"Find Protein Structures"*. The UniChem web service provides database cross-references based on the InChI-Keys returned from PubChem. In the column "Additional compound information" of the result table, links are provided to KEGG (Kanehisa and Goto, 2000), DrugBank (Wishart *et al.*, 2006, 2008), ChEBI (Degtyarenko *et al.*, 2008), ChEMBL (Gaulton *et al.*, 2012), and ZINC (Irwin *et al.*, 2012) databases. To make it easier for the user, the InChI can be used to automatically populate the search box for the E.C. and InChI-based search in the *"Ligand functional annotation"* tool.

Design: The PubChem queries are performed using python to query the PUG interface. Results are passed from the LigDig server to the client browser in JavaScript Object Notation (JSON) format. To avoid cross-site-scripting limitations when accessing UniChem to provide database cross-references, the LigDig web server queries UniChem using the Play framework web services extensions, and the results are passed to a client side JavaScript that updates the web page HTML asynchronously.

2.2 Find an inhibitor

Motivation: Recently there has been much discussion of the impact of polypharmacology on drug discovery efforts. Similar principles are also important for systems biology approaches where a chemical inhibitor can be applied to inhibit a specific component of a network or pathway. Many compounds exhibit polypharmacology meaning that they act on multiple proteins. It would be desirable for a bench scientist to be able to easily discover these proteins. If they find a compound that inhibits multiple proteins within their pathway or network they may then select another more specific inhibitor, or choose to explicitly model this effect.

Functionality: The *"Find an Inhibitor"* tool finds inhibitors of a given protein and then, for each inhibitor, finds all other proteins against which it has activity. To do this, a UniProt identifier is required. The search box on this page has an auto-complete functionality to assist the user, who can provide a full-text string, for which UniProt identifiers are suggested. ChEMBL is then queried once using the supplied UniProt identifier. The resulting compounds that have activity values (IC_{50}, K_d, K_i, Potency, EC_{50}, XC_{50}, AC_{50}) are returned. When more than 100 compounds meet this criterion, only those that exceed a user defined ligand activity threshold value (default 1000 nM) are retained, whereas for fewer than 100 compounds, every compound is returned. The retained compounds are used as queries to find all proteins with activities against them. Results are displayed using Cytoscape.js and additionally in a table. Compounds with activities exceeding the ligand activity threshold value are labelled as potential inhibitors, whereas compounds with activities exceeding a user-defined off-target activity threshold value (default: 100,000 nM) are marked as off-target compounds.

Design: The SQLAlchemy python package is used to interface with the ChEMBL MySQL database files downloaded after each new ChEMBL release (as of June 17th 2014, release 18), which are hosted on a MariaDB SQL server. The query result is rendered into a JSON format suitable for Cytoscape.js. UniChem is used to perform database cross-references for compounds. UniProt identifiers are provided by using the typeahead functionality from Twitter bootstrap 2.3.2 and by querying the local ChEMBL database based on protein name.

2.3 Ligand functional annotation

The ligand functional annotation tool has two possible usage modes: the first uses PDB files as input, the second uses an InChI and an E.C. number. The results for each mode are displayed slightly differently. Both tools are described below.

PDB-based search

Motivation: Ligands that bind to an enzyme can have several functions, e.g., acting as a substrate, product or as different kinds of modifiers, depending on their enzymatic context. If the function of a ligand in a particular receptor-ligand complex structure is not reported as metadata in the PDB database (RCSB), this information must be obtained from other databases or the literature.

© Oxford University Press 2005

Functionality: Starting with a PDB code, the corresponding enzyme names, UniProt identifiers and E.C. numbers, as well as ligand names and SMILES strings of the molecules in the structure, are retrieved from the PDB database. Depending on the user-supplied parameters, the enzyme names, UniProt identifiers and/or E.C. numbers are used as search terms to query the reaction database SABIO-RK. Subsequently, each compound name is used to query the PubChem database, and the structural similarity for all PDB ligand-SABIO-RK compound pairs is analysed by calculating the Tanimoto coefficient. The user is presented with a table containing all ligand:SABIO-RK compound pairs above a user-specified similarity threshold. In addition, compound function, given by an SBO term in SABIO-RK, is supplied.

Design: Python is used to perform RESTful queries of the RCSB PDB. SABIO-RK is also queried using RESTful web services, and results are returned as SBML. SBML is parsed using the python wrappers for libSBML (Bornstein *et al.*, 2008). Subsequently, the SBML files are parsed, and each compound name is used to query the PubChem database for the isomeric SMILES string. For the sake of standardisation, SMILES strings are also requested from PubChem using the SMILES strings of each ligand provided by the PDB database. Using these SMILES strings, the structural similarity for all PDB ligand-SABIO-RK compound pairs is analysed by calculating the Tanimoto coefficient using the FP2 molecular fingerprint implemented in Open Babel, which encodes molecular structures in a series of bits that represent the presence or absence of particular substructures in the molecule.

E.C. and InChI-based search

Motivation: Many enzymes have multiple functionalities, even if the E.C. number does not reveal them all. Knowledge of this functionality can help the user to find out whether a given compound might be involved in a reaction involving a specified E.C. number.

Functionality: The user chooses an E.C. number for the enzyme that they are interested in. The user then supplies an InChI, which can be pasted from an external data source, or pre-populated by using the *"Find Compound by Name"* tool. SABIO-RK is queried to return an SBML file containing all reactions involving the given E.C. number. A list of all products and reactants for which a ChEBI or KEGG identifier exists in SABIO-RK is extracted. To assist the user, a Tanimoto similarity score is calculated for each compound with respect to the query compound. The data are displayed as a table that can be sorted or filtered as required by the user.

Design: The SBML file returned from the SABIO-RK RESTful interface (Wittig *et al.*, 2012) is parsed using the JSBML package for Java (Dräger *et al.*, 2011). A list of all products and reactants for which a ChEBI or KEGG identifier exists in SABIO-RK is extracted and an InChI is returned for each using the UniChem web service (Chambers *et al.*, 2013). The CDK toolkit is used to generate 2D images for each compound, and a table is returned to the user displaying all results. Results are served to the client as JSON and displayed using dynatable.js.

2.4 Batch search for ligand identifier

Motivation: Many compound names used in the literature are stored as a list of synonyms in databases like PubChem or ChEBI. Due to ambiguous naming, database searches using compound names can result in multiple identifiers; therefore, a selection of the correct identifiers must be carried out by hand. This is time consuming and can be error-prone.

Functionality: LigDig enables automatic searching of the PubChem and ChEBI databases, via upload of an Excel file in .xlsx format containing the names of compounds of interest. Furthermore, if the user provides the molecular formula and/or the formal net charge for each compound, the retrieved database compounds are validated against these values by comparing the number of atoms and whether the given formal charge is possible. The highest and lowest formal charges are calculated using either the default pH values 1 and 14, respectively, or a user-specified pH range. In the case of a mismatch with the input molecular formula or a formal charge beyond the calculated range, the selected database compound identifier is not written to the output Excel file.

Design: Validation of a compound against the user-supplied molecular formula and/or net-charge is done by calculating the structural topology, including hydrogens, of the retrieved SMILES string using Open Babel (O'Boyle *et al.*, 2011) in combination with the MMFF94 force field (Halgren, 1996). Excel files are written using the openpyxl python package.

2.5 Find protein structures

Motivation: Many of the tools in LigDig require a PDB code as an input, e.g. *"PDB-based ligand functional annotation"*, *"Binding site comparison"* or *"Structure preparation"*. Therefore we provide a simple way to find protein structures in the PDB . Typically, the user would reach this page by first using the *"Find compound by name"* tool as described previously.

Functionality: The input list of PDB codes can be entered manually from the search page of "Find Protein Structure" or be sent directly from the results page of "Find Compound by Name". The PDB files themselves are not downloaded at this stage. E.C. number, UniProt (Consortium, 2013), SCOP (Murzin *et al.*, 1995) and PFAM (Mistry *et al.*, 2013) identifiers and links are presented for each PDB code in a sortable table.

© Oxford University Press 2005

Design: A list of PDB codes is passed to the RCSB PDB RESTful web services using python.

2.6 Binding site comparison

The binding site comparison tool can be used to align two protein structures by superposing either ligands in the binding site, or amino acid residues that define the binding site.

Ligand-based superposition

Motivation: Ligand-based superposition of binding sites can be useful when two protein structures contain the same ligand or ligands that have similar chemical structures. Comparison of the binding sites relative to the superposed ligand orientation can give important clues about conserved binding residues.

Functionality: The user supplies a list of PDB files containing the ligands in which they are interested. This list can be auto-populated using the previously described *"Find protein structures"* tool. The ligand binding sites, defined by the set of residues that contains at least one atom within a defined distance of any ligand atom (typically 5 Å), are extracted. Ligands are then superposed using the fconv tool (Neudert and Klebe, 2011). The user is then supplied with a JSmol visualization and several UI components to help visualize the binding site.

Design: PDB files are downloaded from the RCSB PDB using the RESTful interface, and components used in the structure preparation tool are reused to extract the ligand, and the binding site from the PDB file. For each binding site, the rotation and translation used to superpose the query ligand onto the reference ligand are computed and applied to the query binding site using a simple python script, in order to visualize the binding sites correctly in relation to the reference ligand. JSmol is used for PDB file visualization.

Residue-based superposition

Motivation: Residue-based binding site superposition not only allows the comparison of binding sites at which similar ligands are known to bind, but it also allows the comparison of arbitrarily defined binding sites for which no ligand is known. Residue-based superposition is particularly useful when similar binding sites bind similar ligands, even though the respective protein folds are quite different.

Functionality: This tool allows the user to find similar binding sites even if they are known to bind to very different ligands. The residue-based binding site superposition mode also allows new binding sites to be found as a ligand is not necessarily required. It makes use of the ProBiS tool (Konc and Janezic, 2010). Structure files do not need to be prepared for using the ProBiS web server, however, protein chains and ligands need to be selected by the user for each PDB file. A table displaying the chain and ligand information to the user is then displayed where the user can select which binding sites to superpose, or which PDB chains to search for similar binding sites. The ProBiS web server returns the superposed structures and further information about the alignment. This enables the user to visualize only the residues considered similar in the local structural alignment.

Design: The underlying ProBiS (Konc and Janezic, 2010) algorithm finds structurally similar protein binding sites and performs pairwise local structural alignments. The RESTful interface of a locally installed ProBiS tool is used to compute the local similarity of the residues around the specified ligands or in the complete protein chains. To assist the user to determine which binding sites to superpose using ProBiS The RESTful web service for the internally available OpenSiteFinder web server returns a JSON file containing the list of proteins and ligands which determine binding sites that could possibly be superposed.

2.7 Structure preparation

Motivation: Most crystal structures of proteins lack hydrogen atoms. The aim of this tool is to add hydrogen atoms to structures of protein-ligand complexes so that the coordinates can be used in further calculations and analyses. The tool cannot currently handle missing non-hydrogen atoms.

Functionality: Starting either with a list of PDB codes or a single PDB file uploaded by the user, hydrogen atoms can be added to receptor and ligand crystal structures using PDB2PQR and fconv, respectively. Due to the limitations of PDB2PQR, metal ions, non-standard amino acids, and covalently bound ligands given in the PDB files used cannot be handled, and are pasted unprocessed to the final downloadable output PDB file. In addition, intermediate files for the receptors and extracted ligands are provided in PDB and mol2 file formats, respectively.

Design: PDB files for the input PDB codes are downloaded automatically from the RCSB PDB or, for obtaining biological units, from the wwPDB. Alternatively, a user-specified PDB file can be uploaded manually for further processing. Metal ions and ligands are written to mol2 files and the latter are protonated using fconv (Neudert and Klebe, 2011). After changing all atom types in the individual mol2 files to the format used in PROPKA (Dolinsky *et al.*, 2007), all ligand files are merged into a single mol2 file. Finally, PDB2PQR (Dolinsky *et al.*, 2004, 2007) is used to add hydrogen atoms to the receptor structure and water molecules. In the case where a bound ligand is contained in the PDB file, the previously described mol2 file suitable for use in PDB2PQR, is also provided. Version 1.8 of PDB2PQR cannot handle metal ions or modified amino acids by default; therefore, missing atoms that

© Oxford University Press 2005

are not processed by PDB2PQR are concatenated to the final pdb file.

3 REFERENCES

Berman, H. M., Battistuz, T., Bhat, T. N., Bluhm, W. F., Bourne, P. E., Burkhardt, K., … Zardecki, C. (2002). The Protein Data Bank. Acta Crystallographica. Section D, Biological Crystallography, 58(6), 899–907. doi:10.1107/S0907444902003451

Bolton, E. E. (2008). Chapter 12 PubChem: Integrated Platform of Small Molecules and Biological Activities (Vol. 4, pp. 217–241). doi:10.1016/S1574-1400(08)00012-1

Chambers, J., Davies, M., Gaulton, A., Hersey, A., Velankar, S., Petryszak, R., … Overington, J. P. (2013). UniChem: a unified chemical structure cross-referencing and identifier tracking system. Journal of Cheminformatics, 5(1), 3. doi:10.1186/1758-2946-5-3

Consortium, T. U. (2013). Update on activities at the Universal Protein Resource (UniProt) in 2013. Nucleic Acids Research, 41(Database issue), D43–7. doi:10.1093/nar/gks1068

Degtyarenko, K., de Matos, P., Ennis, M., Hastings, J., Zbinden, M., McNaught, A., … Ashburner, M. (2008). ChEBI: a database and ontology for chemical entities of biological interest. Nucleic Acids Research, 36(Database issue), D344–50. doi:10.1093/nar/gkm791

Dolinsky, T. J., Czodrowski, P., Li, H., Nielsen, J. E., Jensen, J. H., Klebe, G., & Baker, N. A. (2007). PDB2PQR: expanding and upgrading automated preparation of biomolecular structures for molecular simulations. Nucleic Acids Research, 35(Web Server issue), W522–5. doi:10.1093/nar/gkm276

Dolinsky, T. J., Nielsen, J. E., McCammon, J. A., & Baker, N. A. (2004). PDB2PQR: an automated pipeline for the setup of Poisson-Boltzmann electrostatics calculations. Nucleic Acids Research, 32(Web Server issue), W665–7. doi:10.1093/nar/gkh381

Dräger, A., Rodriguez, N., Dumousseau, M., Dörr, A., Wrzodek, C., Le Novère, N., … Hucka, M. (2011). JSBML: a flexible Java library for working with SBML. Bioinformatics (Oxford, England), 27(15), 2167–8. doi:10.1093/bioinformatics/btr361

Gaulton, A., Bellis, L. J., Bento, A. P., Chambers, J., Davies, M., Hersey, A., … Overington, J. P. (2012). ChEMBL: a large-scale bioactivity database for drug discovery. Nucleic Acids Research, 40(Database issue), D1100–7. doi:10.1093/nar/gkr777

Halgren, T. A. (1996). Merck molecular force field. I. Basis, form, scope, parameterization, and performance of MMFF94. Journal of Computational Chemistry, 17(5-6), 490–519. doi:10.1002/(SICI)1096-987X(199604)17:5/6<490::AID-JCC1>3.0.CO;2-P

Hanson, R. M., Prilusky, J., Renjian, Z., Nakane, T., & Sussman, J. L. (2013). JSmol and the Next-Generation Web-Based Representation of 3D Molecular Structure as Applied to Proteopedia. Israel Journal of Chemistry, 53(3-4), 207–216. doi:10.1002/ijch.201300024

Heller, S., McNaught, A., Stein, S., Tchekhovskoi, D., & Pletnev, I. (2013). InChI - the worldwide chemical structure identifier standard. Journal of Cheminformatics, 5(1), 7. doi:10.1186/1758-2946-5-7

Irwin, J. J., Sterling, T., Mysinger, M. M., Bolstad, E. S., & Coleman, R. G. (2012). ZINC - A free tool to discover chemistry for biology. Journal of Chemical Information and Modeling. doi:10.1021/ci3001277

Kanehisa, M., & Goto, S. (2000). KEGG: kyoto encyclopedia of genes and genomes. Nucleic Acids Research, 28(1), 27–30.

Konc,J. and Janezic,D. ProBiS–2012: web server and web services for detection of structurally similar binding sites in proteins. Nucleic Acids Res., 2012, 40, W214-W221.

Konc,J. and Janezic,D. ProBiS algorithm for detection of structurally similar protein binding sites by local structural alignment. Bioinformatics 2010, 26, 1160-1168.

Li, H., Robertson, A. D., & Jensen, J. H. (2005). Very fast empirical prediction and rationalization of protein pKa values. Proteins, 61(4), 704–21. doi:10.1002/prot.20660

Mistry, J., Coggill, P., Eberhardt, R. Y., Deiana, a., Giansanti, a., Finn, R. D., … Punta, M. (2013). The challenge of increasing Pfam coverage of the human proteome. Database, 2013, bat023–bat023. doi:10.1093/database/bat023

Murzin, A., Brenner, S., Hubbard, T., & Chothia, C. (1995). SCOP: A structural classification of proteins database for the investigation of sequences and structures. Journal of Molecular Biology, 247(4), 536–540. doi:10.1016/S0022-2836(05)80134-2

Neudert, G., & Klebe, G. (2011). fconv: Format conversion, manipulation and feature computation of molecular data. Bioinformatics (Oxford, England), 27(7), 1021–2. doi:10.1093/bioinformatics/btr055

O'Boyle, N. M., Banck, M., James, C. A., Morley, C., Vandermeersch, T., & Hutchison, G. R. (2011). Open Babel: An open chemical toolbox. Journal of Cheminformatics, 3(1), 33. doi:10.1186/1758-2946-3-33

Wishart, D. S., Knox, C., Guo, A. C., Shrivastava, S., Hassanali, M., Stothard, P., … Woolsey, J. (2006). DrugBank: a comprehensive resource for in silico drug discovery and exploration. Nucleic Acids Research, 34(Database issue), D668–72. doi:10.1093/nar/gkj067

© Oxford University Press 2005
